# Identification of biomarkers in patients with rheumatoid arthritis responsive to DMARDs but with progressive bone erosion

**DOI:** 10.3389/fimmu.2023.1254139

**Published:** 2023-09-19

**Authors:** Emiliano Marasco, Gianluigi Fabbriciani, Laura Rotunno, Matteo Longhi, Paola De Luca, Laura de Girolamo, Alessandra Colombini

**Affiliations:** ^1^ Division of Rheumatology, Ospedale Pediatrico Bambino Gesù IRCCS, Roma, Italy; ^2^ Ph.D. Course “Immunology, Molecular Medicine and Applied Biotechnology”, University of Rome Tor Vergata, Rome, Italy; ^3^ Unit of Rheumatology, IRCCS Istituto Ortopedico Galeazzi, Milan, Italy; ^4^ Laboratorio di Biotecnologie Applicate all’Ortopedia, IRCCS Istituto Ortopedico Galeazzi, Milan, Italy

**Keywords:** rheumatoid arthritis, bone erosion, immune cells, cytokines, bone markers

## Abstract

**Introduction:**

Rheumatoid arthritis (RA) is an inflammatory autoimmune disease that may cause joint destruction and disability. The pharmacological treatment of RA aims at obtaining disease remission by effectively ceasing joint inflammation and arresting progressive bone erosions. Some patients present bone lesions accrual even after controlling joint inflammation with current therapies. Our study aimed to analyze lymphocyte subsets and levels of circulating cytokines in patients with RA with progressive bone erosions.

**Methods:**

We enrolled 20 patients with a diagnosis of RA and 12 healthy donors (HD). Patients with RA were divided into patients with bone erosions (RA-BE+) and without bone erosions (RA-BE-). Lymphocyte subsets in peripheral blood were evaluated by flow cytometry. Circulating cytokines levels were evaluated by protein array.

**Results:**

The distribution of lymphocyte subsets was not able to separate HD from AR patients and RA-BE+ and RA-BE- in cluster analysis. We observed a significant expansion of CXCR5^-^ PD1^+^ T peripheral helper cells (Tph cells) and a reduction in both total memory B cells and switched memory B cells in RA patients compared to HD. We observed an expansion in the frequency of total B cells in RA-BE+ patients compared to RA-BE- patients. Unsupervised hierarchical clustering analysis of 39 cytokines resulted in a fairly good separation of HD from RA patients but not of RA-BE+ patients from RA-BE- patients. RA-BE+ patients showed significantly higher levels of IL-11 and IL-17A than RA-BE- patients.

**Conclusion:**

We show that patients with progressive erosive disease are characterized by abnormalities in B cells and in cytokines with a proven role in bone reabsorption. Understanding the role played by B cells and the cytokine IL-11 and IL-17A in progressive erosive disease can help identify novel biomarkers of erosive disease and design treatment approaches aimed at halting joint damage in RA.

## Introduction

Rheumatoid arthritis (RA) is an inflammatory autoimmune disease with a global prevalence of 0.5–1% (1). Disease-modifying anti-rheumatic drugs (DMARDs) are the mainstay of the pharmacological treatment for RA, with methotrexate representing the first-line drug, often combined with low-dose glucocorticoids or other classical DMARDs. If the first therapeutic approach is ineffective, biological or synthetic DMARDs are employed (2).

The pharmacological treatment of RA aims at obtaining disease remission, by effectively ceasing joint inflammation and arresting progressive bone erosion. Nevertheless, some patients present bone lesions accrual even during treatment with a biologic agent. These pathological lesions are the results of structural bone damage in a compromised joint, showing also degradation of articular cartilage and synovitis, with increased vascularization of the synovial membrane and immune cell infiltration (3). In this scenario, there is a close relationship between the dysregulated immune system mediating inflammation and bone metabolism (4). Specifically, immune cells produce pro-inflammatory and bone-resorption-associated cytokines and autoantibodies able to promote bone degradation and contemporarily counteract new bone formation (5, 6).

Experimental and clinical studies to identify the molecular mechanisms underlying RA-associated bone erosion are lacking. The present study aims at discovering novel molecular pathways in RA patients responsive to DMARDs but with progressive bone erosion.

## Materials and methods

### Enrollment of patients and sample collection

Patients with RA fulfilling the 2010 ACR/EULAR criteria (7) were enrolled at the Division of Rheumatology of the IRCCS Istituto Ortopedico Galeazzi (Milan, Italy), after signing the written informed consent (protocol AREOS, approved on 25/10/2017 by Institutional Review Board ASL Città di Milano). Subjects without RA enrolled at the Institute, and before performing ligament repair surgeries, comprised the control cohort. All the subjects were enrolled from February 2018 to March 2021. We screened 28 patients with a diagnosis of RA. Five were excluded for being at RA onset and three were excluded for missing information on clinical history and radiographic images. Of the 20 patients included, 10 presented bone erosion identified by an expert clinician through X-rays (RA-BE+) and 10 showed no bone erosions (RA-BE-). From each enrolled patient, clinical and biochemical laboratory data were collected; whole blood was obtained and used for peripheral blood mononuclear cell (PBMC) isolation and plasma and serum collection.

### Isolation of PBMCs and flow cytometry analysis

PBMCs were isolated from the whole blood by density gradient centrifugation (Ficoll-Hypaque; GE Healthcare, UK), following the manufacturer’s standard procedure, and were cryopreserved until analyzed.

After thawing, 3×10^5^ PBMCs were stained with a mix of antibodies (all from Biolegend, San Diego, California, USA), allowing for the analysis of B, T, and T helper cells. In particular, for B cells: PE-Cy5 anti-human CD19, APC anti-human CD24, PE anti-human CD27, PE-Cy7 anti-human CD38, and FITC anti-human IgD antibodies were used. For T and T helper cells: FITC anti-human CD3, PE-Cy7 anti-human CD4, PerCP anti-human CD8, APC anti-human TCR αβ, PE anti-human TCR γδ, PE anti-human CD185 (CXCR5), and APC anti-human CD279 (PD-1) were used. Flow cytometry data were collected on a Cytoflex flow cytometer (Beckman Coulter Brea). Data were analyzed with FlowJo™ Software version 10.

### Protein array of inflammatory and bone remodeling markers

Quantibody^®^ Human Inflammation Array and Custom Human Quantibody^®^ Array were used to determine the concentration of 38 soluble inflammatory and 9 bone remodeling factors (RayBiotech, Norcross, GA, USA).

### Statistical analysis

All statistical analyses were performed using R (version R 4.0.3) and R studio (RStudio Team, 2020). Descriptive statistics were presented as absolute and relative frequencies and as median with IQR. Data were checked for normality. Parametric tests were used for normally distributed data. For not normally distributed data, non-parametric statistical tests were employed. Tables were generated with the Tableone package. We set significance levels at p<0.05.

Lymphocyte populations and serum cytokines were analyzed by means of unsupervised clustering using the Euclidean distance and the ward.D method for calculating similarities by the hclust and pheatmap R packages.

## Results

### Patient characteristics

Our study enrolled 20 patients with a diagnosis of RA and 12 healthy donors (HD). Patients and HD were matched for age, sex, and body mass index (BMI) (**Supplementary Table 1**). The demographic and clinical characteristics of patients are represented in **Table 1**. A sample of blood was obtained for each patient and control at enrollment. PBMCs and plasma were isolated as described in the methods section. At enrollment, disease activity for patients with RA was defined by the evaluating rheumatologist (**Table 1**). Hands and feet X-rays were retrieved and assessed for the presence of bone erosions. Erosive RA was defined according to the EULAR definition of erosive disease (8): 10 patients had erosive RA (RA-BE+) and 10 patients had non-erosive RA (RA-BE-) (**Table 1**). There were 17 (85%) patients with RA who were in remission or had low disease activity as defined according to SDAI, 2 (10%) patients and 1 (5%) patient were, respectively, in moderate and high disease activity at enrollment. There were 14 (70%) patients who presented anti-citrullinated protein antibodies (ACPA+). Finally, 7 (35%) patients were on methotrexate (MTX) monotherapy, whereas 13 (65%) patients were on biologics; only 3 (15%) patients were on a combination of MTX and biologics. There were no significant differences between RA-BE+ and RA-BE- patients, except for a longer disease duration for RA-BE+ patients (**Table 1**).

**Table 1:** Demographic and clinical characteristics of the patients.

	All (n=20)	RA BE- (n=10)	RA BE+ (n=10)	p-values
**Age, years**	59.5 [53.2-66.5]	65.0 [57.2-67.5]	51.5 [43.0-61.7]	0.088
**Sex (males/females)**	8/12	5/5	3/7	0.648
**BMI, kg/m^2^**	25.64±4.0	26.9±3.2	24.3±4.4	0.151
**RA duration (years)**	8.68±4.52	6.13±3.2	11.2±4.3	0.007*
**Therapy, n° (%)**				0.189
**MTX**	7 (35)	6 (60)	1 (10)	
**MTX, Abatacept**	1 (5)	0 (0)	1 (10)	
**MTX, Adalimumab**	1 (5)	1 (10)	0 (0)	
**MTX, Etanercept**	1 (5)	0 (0)	1 (10)	
**Abatacept**	3 (15)	2 (20)	1 (10)	
**Certolizumab**	1 (5)	0 (0)	1 (10)	
**Etanercept**	3 (15)	1 (10)	2 (20)	
**Sarilumab**	2 (10)	0 (0)	2 (20)	
**Tocilizumab**	1 (5)	0 (0)	1 (10)	
**Scores**				
**TJC**	0.9±1.9	0.8±2.5	1.0±1.1	0.823
**SJC**	0.9±1.9	0.8±2.5	1.0±1.1	0.823
**PGA**	3.2±2.2	4.1±2.1	2.4±2.0	0.08
**EGA**	2.6±2.4	3.3±2.7	2.0±1.8	0.229
**VAS**	30.5±27.0	40.1±22.4	21.0±28.8	0.116
**ESR (mm/h)**	23.0±24.6	32.7±32.5	14.4±9.9	0.108
**CPR (mg/L)**	1.2±1.6	1.6±1.7	0.9±1.5	0.368
**DAS28**	2.3±1.3	2.4±1.7	2.2±0.8	0.842
**SDAI**	8.9±7.8	10.6±9.5	7.3±5.7	0.363
**Disease activity states based on SDAI, n° (%)**				0.196
**High disease**	1 (5)	1 (10)	0 (0)	
**Moderate disease**	2 (10)	0 (0)	2 (20)	
**Low disease**	13 (65)	8 (80)	5 (50)	
**Remission**	4 (20)	1 (10)	3 (30)	
**SENS**			11.0 [3.7-22.7]	
**Antibody positive, n° (%)**				
**ACPA**	14 (70)	8 (80)	6 (60)	0.626
**RF**	14 (77)^f^	7 (87)^f^	7 (70)	0.223

Except where indicated otherwise, values are expressed as mean±SD or median and interquartile range. RA-BE-=non-erosive rheumatoid arthritis; RA-BE+=erosive rheumatoid arthritis; BMI=body mass index; MTX=methotrexate; Tender Joint Count=TJC; Swollen Joint Count=SJC; Patient’s Global Assessment=PGA; Evaluator's Global Assessment=EGA; Visual Analogue Scale=VAS; Disease Activity Score=DAS; Simplified Disease Activity Index=SDAI; Erythrocyte Sedimentation Rate=ESR; C-Reactive Protein=CRP; Simplified Erosion Narrowing Score=SENS; ACPA=anti-citrullinated protein antibody; RF=rheumatoid factor. *p≤0.05.

^f^data not available for 2 patients.

### B and T cell abnormalities in patients with RA

**Figure 1:**
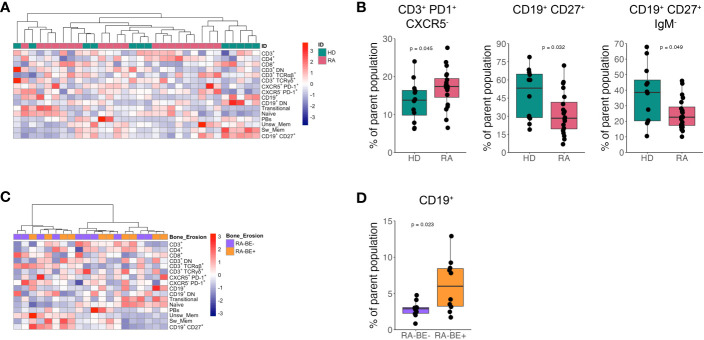
Frequency of lymphocyte subsets in patients with RA and HD. Heatmap of unsupervised clustering using Euclidean distance and ward.D method of lymphocyte subsets in patients with RA (n=20) and HD (n=12) **(A)**. Frequency of CD3^+^ PD1^+^ CXCR5^+^ Tph cells, CD19^+^ CD27^+^ memory B cells and CD19^+^ CD27^+^ IgM^-^ switched memory B cells in RA patients (n=20) and HD (n=12) **(B)**. Heatmap of unsupervised clustering using Euclidean distance and ward.D method of lymphocyte subsets in RA-BE- (n=10) and RA-BE+ patients (n=10) **(C)**. Frequency of CD19^+^ total B cells in RA-BE- (n=10) and RA-BE+ patients (n=10) **(D)**. The p-value is from the Wilcoxon rank-sum test. RA-BE-: RA patients without bone erosions; RA-BE+: RA patients with bone erosions.

We analyzed T and B cell subpopulations in patients and HD by flow cytometry as described in **Supplementary Figure 1**. Unsupervised hierarchical clustering analysis of T and B cell subpopulations resulted in no specific clustering of patients and HD (**Figure 1A**). Data reporting the percentage of T and B cell subpopulations in patients and controls are shown in **Supplementary Tables 2**, **3**.

We observed a significant expansion of CXCR5^-^ PD1^+^ T peripheral helper cells (Tph cells) and a reduction in both total CD27^+^ memory B cells and CD27^+^ IgM^-^ switched memory B cells in patients with RA compared to HD (**Figure 1B**).

We then asked whether lymphocyte subpopulations could differentiate RA-BE+ from RA-BE- patients. Unsupervised hierarchical clustering analysis of T and B cell subpopulations resulted in no specific clustering of RA-BE+ from RA-BE- (**Figure 1C**). Of all the lymphocyte subpopulations analyzed, we could observe an expansion in the frequency of total CD19^+^ B cells in RA-BE+ patients compared to RA-BE- patients (**Figure 1D**).

### Circulating cytokines levels in patients with RA

**Figure 2:**
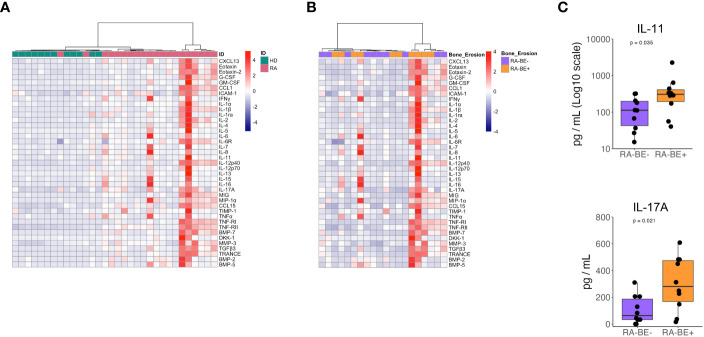
Levels of cytokines in patients with RA and HD. Heatmap of unsupervised clustering using Euclidean distance and ward.D method of circulating cytokines in RA patients and HD **(A)**. Heatmap of unsupervised clustering using Euclidean distance and ward.D method of circulating cytokine levels in RA-BE- and RA-BE+ patients **(B)**. Plasma levels of IL-11 and IL-17A in RA-BE- (n=10) and RA-BE+ patients (n=10) **(C)**. The p-value is from the Wilcoxon rank-sum test. RA-BE-: RA patients without bone erosions; RA-BE+: RA patients with bone erosions.

We analyzed plasma levels of 47 cytokines in patients and HD: 38 inflammatory cytokines and 9 bone-related cytokines. Of these, 39 cytokines showed significantly higher levels in patients than HD (**Supplementary Table 4**). The 8 cytokines that were not significantly different between patients and HD were excluded from the following analysis. Unsupervised hierarchical clustering analysis of 39 cytokines resulted in a fairly good separation of HD from RA patients: only four RA patients clustered in cluster 1 with all HD (**Figure 2A**).

When comparing RA-BE+ and RA-BE- patients with unsupervised hierarchical clustering, our panels of 39 cytokines did not separate the two groups into different clusters (**Figure 2B**). When analyzing the 39 cytokines separately, we found that RA-BE+ patients showed significantly higher levels of IL-11 and IL-17A than RA-BE- patients (**Figure 2C**, **Supplementary Table 5**). We correlated levels of IL-11 and IL-17A with the Simplified Erosion Narrowing Score (SENS), which quantifies the entity of bone erosions: we did not observe any significant correlation between IL-11, IL-17A, and SENS.

## Discussion

The main findings of our study are that patients with RA have abnormalities in the distribution of lymphocytes: we observed an expansion of Tph cells and a reduction in the frequency of memory cells in patients with RA. When comparing patients with BE to patients without BE, we observed that RA patients with BE showed a higher frequency of total CD19^+^ B cells. Patients with RA showed higher levels of circulating pro-inflammatory cytokines and mediators associated with bone reabsorption. Interestingly, RA patients with BE showed higher levels of IL-11 and IL-17A compared to patients without BE.

Bone erosion is one of the central features of RA and it is a consequence of the crosstalk between immune cells and inflammatory mediators and the bone tissue adjacent to the inflamed synovium (9). Bone erosion is defined as an interruption in the cortical bone surface accompanied by loss of the adjacent trabecular bone. Erosions are a negative prognostic factor in RA. They reflect a more severe disease course since they can progress to joint destruction, determining significant disability (6). In consequence of this, the goal of RA treatment is to abate inflammation, in order to control joint pain and improve joint functionality. Moreover, treatment has been shown to limit the occurrence of bone erosions and, thus, damage. The interaction between osteoclasts and the immune system is central to the formation of bone erosions. Therefore, controlling inflammation and the autoimmune response is key to preventing bone erosions.

B cells have a central role in the pathogenesis of RA: they produce ACPA, they can present antigens to T cells, and they secrete pro-inflammatory cytokines. B cells infiltrate the RA synovium, accumulating in tertiary lymphoid tissues, where, with the help of Tph (10), they are activated and refine their antigen receptor in germinal center-like structures (11). B cell depletion with rituximab is effective in treating RA, highlighting their central role in this disease. Moreover, patients treated with TNF inhibitors (12), showed a reduced frequency of tertiary lymphoid tissues in the synovium and a reduced frequency of memory cells in blood and lymphoid organs (13). The presence of APCA is associated with a more aggressive disease progression and ACPA-positive patients are more likely to develop BE (14). B cells play multiple roles in BE; they express RANKL (15, 16) directly activating osteoclasts, and ACPAs can bind to citrullinated vimentin expressed on the surface of osteoclast precursors, promoting bone reabsorption (17). ACPA-positive RA patients present a reduced bone mineral density compared to ACPA-negative RA patients, highlighting the role of ACPAs in bone reabsorption (18). It is interesting to speculate that the higher frequency in total B cells observed in RA patients with BE reflects a general hyperactivation of B cells and, thus, these cells may have a role in the progression of BE.

IL-17 is one of the cytokines involved in bone resorption in RA (19). IL-17 is a family of pro-inflammatory mediators present in synovial joints and produced by Th17 cells. These cytokines act in both early and established RA and contribute to osteoclastogenesis (20, 21), activating fibroblast-like synoviocytes, and recruiting and activating neutrophils, macrophages, and B cells (22). The IL-17 receptor (IL-17R) is present in fibroblasts, epithelial cells, B and T lymphocytes, monocytes, and bone marrow stromal cells (22–24). In RA, serum and tissue levels of IL-17 were shown to be increased (23). Accordingly, we also observed increased circulating levels of IL-17A in our RA cohort. Interestingly, we observed that the levels of this cytokine were significantly higher in RA patients with BE than in RA patients without BE. This result is of particular interest since IL-17 is known to induce the differentiation of osteoclasts, either directly or indirectly through upregulation of RANKL (23). In the inflamed synovial membrane of RA patients, an increased RANKL/OPG ratio promotes osteoclast differentiation and activation at the synovium–bone interface and the development of bone erosions (25, 26). Clinical trials and a systematic review confirmed the efficacy of anti-IL-17 or IL-17R agents in the improvements of RA signs and symptoms (27–34), but no data is available on radiographic progression. It would be interesting to investigate the ability of this class of drugs to control bone erosions.

We observed increased levels of IL-11 in RA patients with BE compared to RA patients without BE. IL-11 belongs to the IL-6 family of cytokines and its contribution to inflammatory diseases, particularly RA, is still unclear. Different studies and clinical trials showed opposing results regarding the role of IL-11 in RA pathogenesis (35). In this context, it is important to underline that IL-11 is involved in bone remodeling (36) by acting on osteoblasts, osteocytes, and osteoclasts (37). Specifically, several groups showed a role of IL-11 in osteoclastogenesis (38) by acting on cells of the hematopoietic lineage (37) and mediating the survival of osteoclast progenitor cells (39). Our results suggest a role of IL-11 in the development of BE and further studies should be designed to better understand the biological mechanisms of IL-11 in BE in RA. Moreover, this cytokine could represent a promising new therapeutic target for RA treatment, especially in patients with progressive erosive disease.

Our study presents some limitations that mainly concern the number and clinical features of the enrolled RA patients. Although we observed statistically significant differences in cytokine levels and lymphocyte subsets between RA patients with and without BE, our cohort was small, and we may have missed other biologically relevant mediators. As a matter of fact, levels of DKK-1, a regulator of osteoblast biology, were higher in RA patients with BE than without BE, but this difference did not reach statistical significance. Other limitations are disease severity, disease duration, and treatment. These represent important confounding factors, especially for investigating immunological biomarkers (40). Cytokines and lymphocytes have been shown to vary between patients with active disease and remission (40). In our study, the majority of RA patients were in remission or low disease activity, with only 15% of patients with active disease distributed between the two experimental groups, making a fairly homogenous cohort. The differences in the disease duration and in the use of biological agents in the experimental groups of our study represent an objective limitation. Longer disease duration is associated with more erosive disease (41); thus, it is not surprising that patients with BE have a longer disease duration than patients without BE. Studies on larger cohorts of patients with RA are needed to replicate and expand the role of B cells and IL-11, IL-17A, and other cytokines as mediators of BE progression in RA. Moreover, only longitudinal studies will elucidate the role of these immunological mediators in bone erosions from disease onset and during follow-up. Overall, our data suggest that patients with progressive erosive disease are characterized by abnormalities in B cells and in cytokines with a proven role in bone reabsorption. Understanding the molecular mechanisms played by B cells and the cytokine IL-11 and IL-17A in progressive erosive disease can help in designing novel treatment approaches aimed at halting joint damage and disability in RA.

## Data availability statement

The raw data supporting the conclusions of this article are available at the following link: https://osf.io/p74q9/?view_only=43aa823384324b0e827ad9a056db17c8.

## Ethics statement

The studies involving humans were approved by Institutional Review Board ASL Città di Milano. The studies were conducted in accordance with the local legislation and institutional requirements. The participants provided their written informed consent to participate in this study.

## Author contributions

EM: Conceptualization, Formal Analysis, Investigation, Methodology, Software, Writing – original draft, Writing – review & editing. GF: Conceptualization, Data curation, Investigation, Writing – review & editing. LR: Data curation, Investigation, Writing – review & editing. ML: Data curation, Investigation, Writing – review & editing. Pd: Data curation, Investigation, Writing – review & editing. Ld: Data curation, Formal Analysis, Funding acquisition, Investigation, Writing – original draft, Writing – review & editing. AC: Conceptualization, Data curation, Formal Analysis, Funding acquisition, Investigation, Methodology, Writing – original draft, Writing – review & editing.
