# Supplementary material for: Identification of biomarkers in patients with rheumatoid arthritis responsive to DMARDs but with progressive bone erosion
Source: Front Immunol. 2023 Sep 19;14:1254139. doi: 10.3389/fimmu.2023.1254139 (PMC10551039; doi:10.3389/fimmu.2023.1254139)
Supplement: Supplementary file 1 [file DataSheet_1.pdf]

Supplementary Table 1. Characteristics of matched patients and controls.

	**RA**<br>(n=20)	**HD**<br>(n=12)
**Age, years**	59.5 [53.2-66.5]	52.0 [45.5-55.7]
**Sex (males/females)**	8/12	5/7
**BMI, kg/m^2**	25.5 [23.4-28.6]	23.4 [22.7-24.3]

Except where indicated otherwise, values are expressed as median and interquartile range.

RA=rheumatoid arthritis patients; HD=healthy donors; BMI=body mass index.

Supplementary Table 2. Percentage of T and B cell subpopulations in RA patients and HD.

	**RA**<br><br>(n=20)	**HD**<br><br>(n=12)	**p-values**
$CD3^+$	77.1 [66.8-81.7]	83.0 [72.1-88.2]	0.186
$CD4^+$	62.2 [57.5-67.6]	67.0 [60.6-71.0]	0.228
$CD8^+$	31.5 [24.6-36.8]	26.7 [23.5-27.7]	0.073
$CD3^+$ DN	6.00 [3.60, 7.58]	5.36 [3.24, 7.90]	0.953
$CD3^+$ $TCR\alpha\beta^+$	72.4 [66.8-81.3]	70.7 [67.1-75.1]	0.312
$CD3^+$ $TCR\gamma\delta^+$	4.2 [2.5-6.1]	2.9 [1.2-6.6]	0.533
$CXCR5^+$ $PD\text{-}1^+$	3.7 [2.1-5.7]	2.7 [1.7-4.6]	0.276
$CXCR5^-$ $PD\text{-}1^+$	17.4 [14.5-19.5]	13.8 [9.8-16.4]	0.043*
$CD19^+$	3.1 [2.5-5.5]	5.5 [3.5-8.6]	0.228
$CD19^+$ DN	6.1 [4.9-7.5]	6.7 [3.6-10.4]	0.785
Transitional	0.8 [0.2-1.4]	0.3 [0.0-0.8]	0.093
Naïve	48.3 [30.7-62.5]	28.2 [17.7- 54.6]	0.129
PBs	0.4 [0.1-0.9]	0.44 [0.19, 0.87]	0.938
Unsw_Mem	9.3 [5.8-19.3]	13.5 [11.4-16.1]	0.235
Sw_Mem	22.6 [17.3-29.2]	38.5 [20.3-46.5]	0.047*
$CD19^+$ $CD27^+$	28.3 [19.5-41.6]	53.2 [28.9-64.8]	0.032*

Values are expressed as median and interquartile range. RA=rheumatoid arthritis patients; HD=healthy donors. *$p \leq 0.05$.

Supplementary Table 3. Percentage of T and B cell subpopulations in RA non-erosive or erosive patients.

	**RA BE-** (n=10)	**RA BE+** (n=10)	**p-values**
$CD3^+$	77.9 [74.2-85.5]	76.1 [66.1-80.2]	0.449
$CD4^+$	62.2 [58.7-69.5]	63.05 [55.68, 67.10]	0.623
$CD8^+$	32.5 [24.7-35.9]	30.5 [25.0-36.6]	0.940
$CD3^+$ DN	4.24 [3.07, 7.32]	7.16 [5.34, 8.69]	0.199
$CD3^+$ $TCR\alpha\beta^+$	78.0 [69.7-82.8]	69.8 [66.1-76.4]	0.174
$CD3^+$ $TCR\gamma\delta^+$	3.4 [2.7-6.0]	4.9 [2.8-6.1]	0.623
$CXCR5^+$ $PD\text{-}1^+$	4.5 [2.41, 5.62]	2.96 [2.00, 5.65]	0.705
$CXCR5^-$ $PD\text{-}1^+$	17.5 [17.0-19.0]	16.9 [13.2-19.9]	0.705
$CD19^+$	2.9 [2.3-3.1]	6.0 [3.2-8.4]	0.023*
$CD19^+$ DN	7.1 [6.4-8.6]	5.0 [4.6-5.9]	0.130
Transitional	0.7 [0.3-1.2]	0.9 [0.3-1.8]	0.496
Naïve	48.6 [33.0-58.7]	48.3 [29.9-68.7]	0.821
PBs	0.5 [0.1-0.8]	0.2 [0.1-1.1]	1.000
Unsw_Mem	10.3 [6.3-19.0]	9.1 [4.8-19.1]	0.880
Sw_Mem	22.0 [18.1-24.8]	30.2 [16.6-36.6]	0.212
$CD19^+$ $CD27^+$	27.7 [23.7-33.5]	34.5 [17.1-51.6]	0.650

Values are expressed as median and interquartile range. RA=rheumatoid arthritis patients; BE-=non-erosive; BE+=erosive. *p≤0.05.

Supplementary Table 4. Plasma levels of cytokines in RA patients and HD.

	RA (n=20)	HD (n=12)	p-value
BLC	58.0 [17.8-188.4]	5.9 [1.7-17.2]	0.001*
Eotaxin	362.1 [215.9-1212.1]	120.5 [87.0-174.5]	0.004*
Eotaxin-2	567.5 [250.3-1508.6]	164.1 [93.8-306.8]	0.007*
G-CSF	309.0 [74.3-1931.0]	7.1 [2.1-21.8]	<0.001*
GM-CSF	89.3 [63.6-229.2]	25.7 [4.1-45.4]	0.001*
I-309	2037.4 [698.7-7619.7]	402.0 [266.7-571.0]	0.002*
ICAM-1	9905.3 [6896.5-11324.2]	6739.2 [6194.8-7052.3]	0.008*
IFNγ	61.2 [23.0-221.6]	7.5 [5.9-30.1]	0.004*
IL-1α	236.4 [184.4-339.5]	87.8 [51.6-137.7]	<0.001*
IL-1β	275.9 [58.6-1291.5]	4.1 [1.7-10.2]	<0.001*
IL-1Ra	257.1 [147.8-693.7]	95.1 [61.3-136.7]	0.002*
IL-2	48.8 [17.4-157.3]	0.0 [0.0-7.0]	<0.001*
IL-4	287.8 [68.7-553.7]	9.6 [0.0-24.5]	0.002*
IL-5	94.0 [25.6-176.5]	21.9 [1.3-30.5]	0.01*
IL-6	24.6 [10.6-75.0]	3.3 [0.1-14.2]	0.013*
IL-6R	3494.5 [3207.3-5029.0]	2868.0 [2694.2-3044.4]	0.001*
IL-7	151.1 [82.6-314.4]	52.0 [20.0-120.1]	0.02*
IL-8	12.6 [5.1-31.6]	2.2 [0.2-7.0]	0.008*
IL-10	16.2 [5.7-21.9]	6.5 [3.5-20.4]	0.311
IL-11	192.7 [64.3-322.2]	6.7 [0.0-60.0]	<0.001*
IL-12p40	110.2 [61.8-467.9]	28.4 [19.4-45.8]	<0.001*
IL-12p70	5.7 [1.5-13.7]	0.0 [0.0-0.3]	0.003*
IL-13	4.6 [2.2-9.8]	1.4 [0.0-2.6]	0.007*
IL-15	27.1 [7.7-54.2]	1.9 [0.2-8.4]	0.003*
IL-16	276.6 [92.7-534.0]	37.0 [27.4-93.2]	0.002*
IL-17A	176.9 [35.5-310.9]	12.7 [0.0-44.2]	0.001*
MCP-1	116.4 [85.2-169.2]	112.7 [84.6-137.9]	0.669
MCSF	11.0 [1.9-32.9]	3.6 [1.7-5.4]	0.185
MIG	1359.6 [70.1-3454.1]	12.5 [1.4-35.2]	<0.001*
MIP-1a	677.4 [319.1-2803.5]	85.0 [52.5-123.6]	<0.001*
MIP-1b	12.5 [8.8-26.4]	9.8 [6.3-12.8]	0.056
MIP-1d	472.9 [273.7-571.7]	191.4 [171.2-221.0]	<0.001*
RANTES	7772.4 [4993.3-9813.8]	6007.7 [5545.2-6814.6]	0.508
TIMP-1	7465.3 [7090.2-7998.6]	6865.9 [6665.1-7171.2]	0.009*
TNFα	24.9 [7.5-57.0]	3.1 [0.0-5.5]	0.002*
TNFβ	112.7 [24.9-312.9]	10.3 [4.0-101.7]	0.086

TNF-RI	15703.6 [8074.5-79661.8]	3515.3 [3021.0-4243.4]	<0.001*
TNF-RII	20239.4 [14417.2-30965.3]	7451.1 [7004.3-8854.4]	<0.001*
BMP-7	716.7 [136.5-2854.1]	42.1 [23.2-124.9]	0.006*
BMP-9	2.0 [1.3-5.1]	1.5 [1.1-2.3]	0.115
DKK-1	5.5 [0.0-10.2]	0.0 [0.0-0.1]	0.002*
MMP-3	5468.6 [4333.4-7300.2]	3827.9 [2710.2-4258.4]	0.002*
PDGF-BB	655.2 [417.0-934.9]	490.3 [360.9-595.8]	0.119
TGFβ3	608.4 [34.0-1463.9]	0.0 [0.0-33.1]	0.003*
TRANCE	3894.8 [637.5-14957.0]	86.5 [0.0-287.2]	0.003*
BMP-2	12.6 [1.7-44.6]	0.0 [0.0-1.9]	0.009*
BMP-5	707.8 [16.9- 2675.9]	0.0 [0.0-38.3]	0.012*

Values are expressed as median and interquartile range. RA=rheumatoid arthritis patients;

HD=healthy donors. *p≤0.05.

Supplementary Table 5. Plasma levels of cytokines in RA non-erosive or erosive patients.

	RA BE-<br>(n=10)	RA BE+<br>(n=10)	p-values
BLC	22.8 [12.3-104.7]	126.6 [41.1-285.8]	0.082
Eotaxin	336.5 [162.1-740.9]	567.0 [258.7-2537.7]	0.545
Eotaxin-2	552.9 [307.1-1314.1]	643.4 [177.6-2726.6]	0.88
G-CSF	309.0 [84.2-817.9]	659.1 [88.0-2370.2]	0.545
GM-CSF	81.6 [63.9-103.6]	114.8 [66.4-359.6]	0.406
I-309	2037.4 [938.9-4022.8]	1846.1 [664.7-11230.3]	0.762
ICAM-1	9524.8 [6848.8-10278.1]	10095.4 [7167.5- 12817.1]	0.364
IFNγ	58.7 [27.5-111.3]	164.8 [20.1-555.2]	0.29
IL-1α	229.8 [198.6-255.4]	254.4 [149.2-596.1]	0.545
IL-1β	238.0 [49.1-837.9]	377.2 [85.1-1490.9]	0.406
IL-1Ra	204.0 [143.9-267.2]	613.4 [182.6-1365.7]	0.082
IL-2	63.2 [20.2-100.9]	44.4 [18.0-623.7]	0.762
IL-4	257.8 [56.2-345.1]	417.4 [130.9-1373.7]	0.226
IL-5	56.5 [32.3-131.5]	152.1 [24.3-266.2]	0.545
IL-6	17.2 [9.9-36.2]	62.2 [16.3-216.4]	0.151
IL-6R	3499.4 [3271.1-4121.0]	3483.3 [3225.6-5571.3]	0.88
IL-7	114.9 [78.5-266.9]	275.3 [106.6-562.3]	0.326
IL-8	9.6 [5.1-17.0]	16.4 [6.1-127.3]	0.326
IL-10	16.2 [9.8-21.1]	12.9 [3.2-22.5]	0.45
IL-11	112.4 [44.2-198.9]	307.8 [200.0-410.8]	0.034*
IL-12p40	91.1 [65.1-207.9]	278.25 [69.2-563.5]	0.364
IL-12p70	4.0 [1.1-8.2]	10.5 [2.8-36.1]	0.162
IL-13	3.4 [2.3-6.6]	6.15 [2.6-24.1]	0.45
IL-15	23.3 [8.43-32.1]	40.6 [10.4-184.4]	0.226
IL-16	222.8 [115.6-373.8]	375.2 [93.5-1861.4]	0.496
IL-17A	63.1 [32.4-186.6]	280.85 [168.0-474.5]	0.019*
MCP-1	116.4 [94.8-126.3]	122.7 [76.9-335.1]	0.65
MCSF	11.3 [2.0-28.4]	11.0 [2.6-213.7]	0.734
MIG	573.6 [106.4-1813.3]	2031.8 [307.5-4238.5]	0.174
MIP-1a	561.5 [213.9-1860.1]	1613.7 [462.1-2972.3]	0.174
MIP-1b	12.5 [10.3-24.0]	13.8 [8.6-33.9]	0.762
MIP-1d	391.6 [219.6-526.2]	499.6 [367.9-659.2]	0.199
RANTES	6806.3 [4620.4-9343.0]	7772.4 [5416.2-13714.8]	0.406
TIMP-1	7429.9 [7017.9-7749.0]	7581.9 [7143.2-8245.0]	0.597
TNFα	22.3 [9.4-31.2]	47.6 [10.7-152.8]	0.257
TNFβ	82.4 [31.4-288.6]	124.3 [20.4-499.1]	0.880
TNF-RI	15703.6 [11834.4-37650.1]	14191.8 [7691.4-92108.8]	1.000

TNF-RII	17355.3 [13596.4-24423.8]	23643.1 [16785.1-38307.2]	0.151
BMP-7	585.3 [115.7-2227.3]	1916.0 [153.3-4881.2]	0.427
BMP-9	1.9 [1.1-3.5]	3.3 [1.8-11.1]	0.185
DKK-1	1.9 [0.0-6.4]	6.7 [5.2-11.2]	0.085
MMP-3	5033.8 [4328.9-7071.2]	6056.0 [5119.3-7220.1]	0.406
PDGF-BB	720.2 [383.0-1106.2]	604.4 [450.7-735.2]	0.762
TGFβ3	397.8 [63.3-1055.1]	830.9 [136.0-3011.0]	0.286
TRANCE	3894.8 [627.0-13093.0]	5755.4 [810.2-37322.9]	0.569
BMP-2	11.1 [0.0-40.3]	13.0 [3.4-48.6]	0.426
BMP-5	273.2 [0.0-1967.0]	1224.1 [422.7-5376.5]	0.128

Values are expressed as median and interquartile range []. RA=rheumatoid arthritis patients;

BE-=non-erosive; BE+=erosive. *p≤0.05.

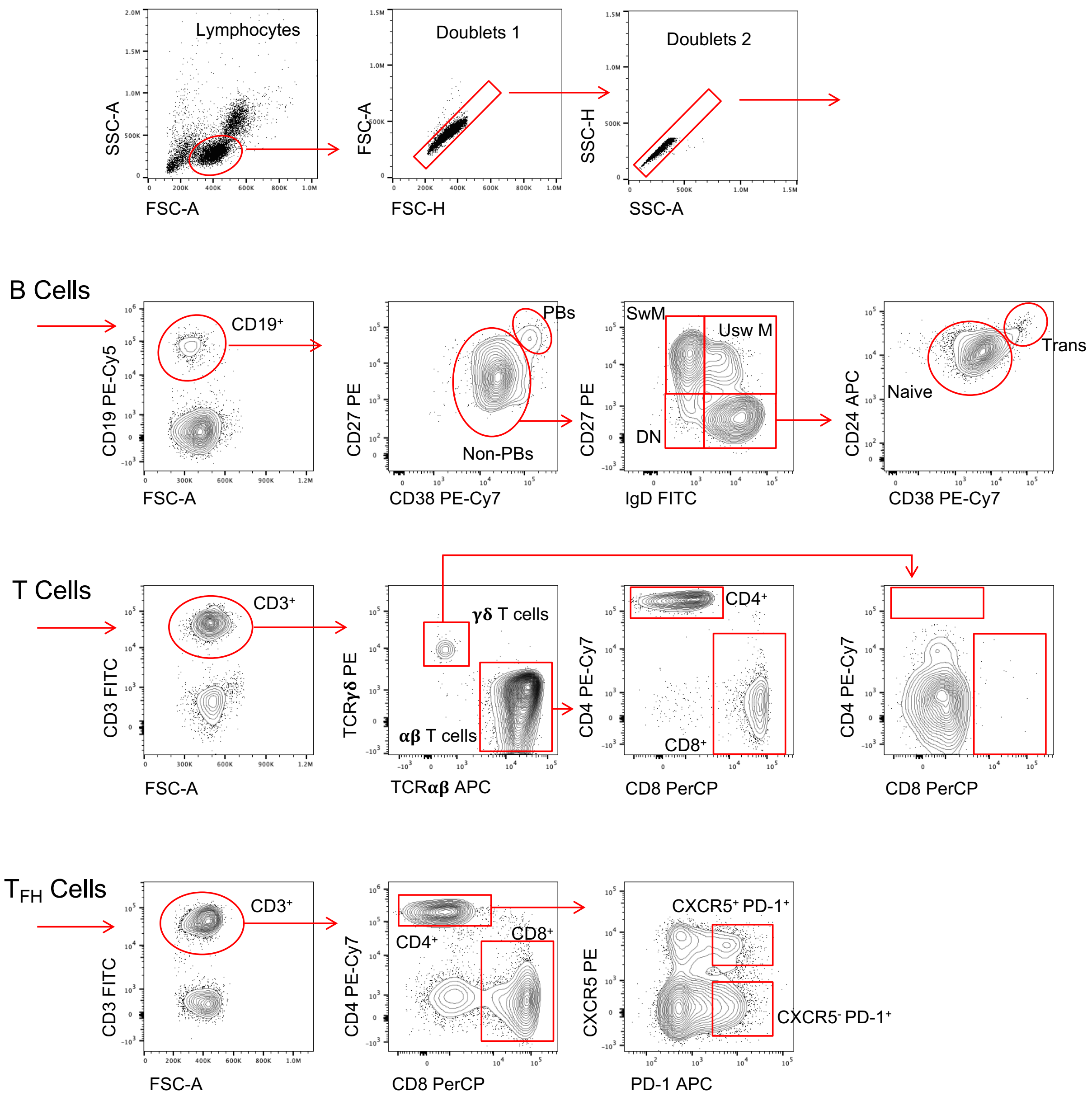

Supplementary Figure 1. Gating strategy of B cell subsets, T cell subsets and T_{FH} cells. DN, double negative B cells, PBs, plasmablasts; SwM, switched memory B cells; Trans, transitional B cells; UswM, unswitched memory B cells.
